# Use of Competition ELISA for Monitoring of West Nile Virus infections in Horses in Germany

**DOI:** 10.3390/ijerph10083112

**Published:** 2013-07-24

**Authors:** Ute Ziegler, Joke Angenvoort, Christine Klaus, Uschi Nagel-Kohl, Claudia Sauerwald, Sabine Thalheim, Steffen Horner, Bettina Braun, Susanne Kenklies, Judith Tyczka, Markus Keller, Martin H. Groschup

**Affiliations:** 1 Friedrich-Loeffler-Institut, Federal Research Institute for Animal Health, Institute of Novel and Emerging Infectious Diseases, Südufer 10, D-17493 Greifswald-Insel Riems, Germany; E-Mails: ute.ziegler@fli.bund.de (U.Z.); joke.angenvoort@fli.bund.de (J.A.); markus.keller@fli.bund.de (M.K.); 2 Friedrich-Loeffler-Institut, Federal Research Institute for Animal Health, Institute of Bacterial Infections and Zoonoses, Naumburger Str. 96a, D-07743 Jena, Germany; E-Mail: christine.klaus@fli.bund.de; 3 Lower Saxony State Office for Consumer Protection and Food Safety (LAVES), Food and Veterinary Institute Braunschweig/Hannover, Institute Hannover, Eintrachtweg 17, D-30173 Hannover, Germany; E-Mail: uschi.nagel-kohl@laves.niedersachsen.de; 4 Landesbetrieb Hessisches Landeslabor, Veterinary virology and molecular biology, Schubertstr. 60, D-35392 Gießen, Germany; E-Mail: Claudia.Sauerwald@lhl.hessen.de; 5 Berlin-Brandenburg State Laboratory (LLBB), Gerhard-Neumann-Str. 2-3, D-15236 Frankfurt (Oder), Germany; E-Mail: Sabine.Thalheim@Landeslabor-bbb.de; 6 Thuringian State Authority for Consumer Protection (TLV), Tennstedter Straße 8/9, D-99947 Bad Langensalza, Germany; E-Mail: Steffen.Horner@tlv.thueringen.de; 7 Landesuntersuchungsamt Rhineland-Palatinate, Blücherstr. 34, D-56073 Koblenz, Germany; E-Mail: Bettina.Braun@lua.rlp.de; 8 State Institute for Consumer Protection of Saxony-Anhalt, Department for Veterinary Medicine, Haferbreiter Weg 132-135, D-39576 Stendal, Germany; E-Mail: susanne.kenklies@lav.ms.sachsen-anhalt.de; 9 State Institute for Chemical and Veterinary Analysis, Weißenburger Str. 3, D-76187 Karlsruhe, Germany; E-Mail: Judith.Tyczka@mlr.bwl.de

**Keywords:** West Nile virus, neutralization, ELISA, serology, horses, Germany, tick-borne encephalitis virus, flavivirus

## Abstract

West Nile virus (WNV) is a mosquito-borne viral pathogen of global importance and is considered to be the most widespread flavivirus in the World. Horses, as dead-end hosts, can be infected by bridge mosquito vectors and undergo either subclinical infections or develop severe neurological diseases. The aim of this study was to detect WNV specific antibodies in horses in Germany as an indicator for an endemic circulation of WNV. Sera from more than 5,000 horses (primarily fallen stock animals) were collected in eight different federal states of Germany from 2010 to 2012. Sera were screened by a competitive ELISA and positive reactions were verified by an indirect IgM ELISA and/or by virus neutralization tests (VNT) for WNV and Tick-borne encephalitis virus (TBEV) in order to exclude cross-reacting antibody reactions. In essence WNV specific antibodies could not be detected in any of the horse sera. Not surprisingly, a small number of sera contained antibodies against TBEV. It is noteworthy that equine sera were often collected from horse carcasses and therefore were of poor quality. Nonetheless, these sera were still suitable for WNV ELISA testing, *i.e.*, they did not produce a high background reaction which is a frequently observed phenomenon. According to these data there is no evidence for indigenous WNV infections in horses in Germany at present.

## 1. Introduction

West Nile virus (WNV) is considered to be the most widespread flavivirus in the World and has been reported from all continents, except for Antarctica [1,2,3,4]. WNV is an arbovirus that is transmitted in an enzootic cycle between ornithophilic mosquitoes and certain wild bird species. WNV infections have been described in a wide variety of vertebrates, and the virus has been found in more than 150 species of wild and domestic birds [5]. The composition of bird and mosquito species [6] differs between the geographical regions where WNV occurs. Most of the susceptible vertebrates do not develop sufficient viremia levels to support virus transmission and are therefore considered as dead-end hosts [7,8]. Infection is usually asymptomatic and especially humans and horses can develop disease as a cause of WNV infection, ranging from a mild febrile illness (West Nile fever) to encephalitis with fatal outcome [7,9,10]. The virus was first isolated in Uganda in 1937 and its epidemiology is continuously changing [11]. In Europe, a variety of WNV strains of diverse virulence, belonging either to lineage 1 or to lineage 2, has been isolated to date. Many WNV outbreaks occurred during the past decade, and since 2008 the virus is heavily spreading throughout central and south-eastern Europe, constituting a serious veterinary and public health problem [2].

In several European countries with WNV outbreaks, e.g., Italy, Spain and Greece, it has been reported that neuroinvasive human cases were probably associated with equine cases, which were linked potentially with infected mosquito vectors [10,12,13]. As WNV disease of horses is often closely connected with human disease, monitoring of the horse population can be a useful diagnostic tool. At the same time, it can provide important information on virus prevalence and can be a helpful tool for investigating the transmission of WNV in an infected area by giving a recommendation concerning human exposure. Therefore equines recently have often been used as indicators to monitor the circulation of WNV in the environment [14,15,16,17]. However, it must be taken into account that sentinels work only in areas without vaccination, as infection derived antibodies cannot be discriminated from vaccination derived antibodies by using of the currently available tests and vaccines. On the other hand, in regions where horses are vaccinated, serological monitoring of sentinel mules or donkeys for WNV may also be a useful tool [18].

A major problem in WNV serology is the cross-reactivity between the various members of the genus *Flavivirus* which can strongly affect serological testing and definite diagnosis of WNV infections. It is well-known that the commercial WNV ELISA used in our study shows a high degree of cross-reactivity with other flaviviruses [17,19]. There are not only serological cross-reactions amongst the members of the Japanese encephalitis virus serogroup, but also cross-reactions to viruses of other groups, such as the tick-borne encephalitis virus (TBEV) serogroup. TBEV occurs in natural foci, and is endemic to many countries in Europe and parts of central and eastern Asia [20].

TBEV infections in equines are usually asymptomatic, but rare exceptions occur. Waldvogel *et al.* first described tick-borne encephalitis in a horse in Switzerland which showed signs of central nervous symptoms [21]. Recently, such TBEV infected horses have also been detected in a routine examination of 130 horse sera from 13 herds in Thuringia [22]. One horse, which originated from a holding in Bavaria, also developed clinical signs that may have been caused by a tick-borne encephalitis virus infection. Hence, covert TBEV infections in horses can play a role in the serological investigation of antibodies against flaviviruses. As WNF in horses is a notifiable disease in Germany, cross-reacting antibodies causing false positive ELISA results may lead to unjustified consequences. Therefore a strategy to discriminate serological cross reactions of WNV with other members of the *Flavivirus* group is essential.

Against the background of the current situation of equine infectious anemia (EIA) and the occurrence of WNV cases in other EU member states a risk-based monitoring approach based primarily on the sampling of deceased horses in rendering plants for animal by-products was carried out. In this study more than 5,000 blood samples were collected from horses in eight different federal states from 2010 to 2012. The aim of this study was to detect WNV specific antibodies in horses in Germany as an indicator for endemic circulation of WNV. Furthermore this study was an additional demonstration that the commercial WNV ELISAs show a high degree of cross-reactivity with other flaviviruses. Positive ELISA reactions were verified by flavivirus-specific neutralization assays to reveal occasional TBEV infections in equines.

## 2. Materials and Methods

**Figure 1:**
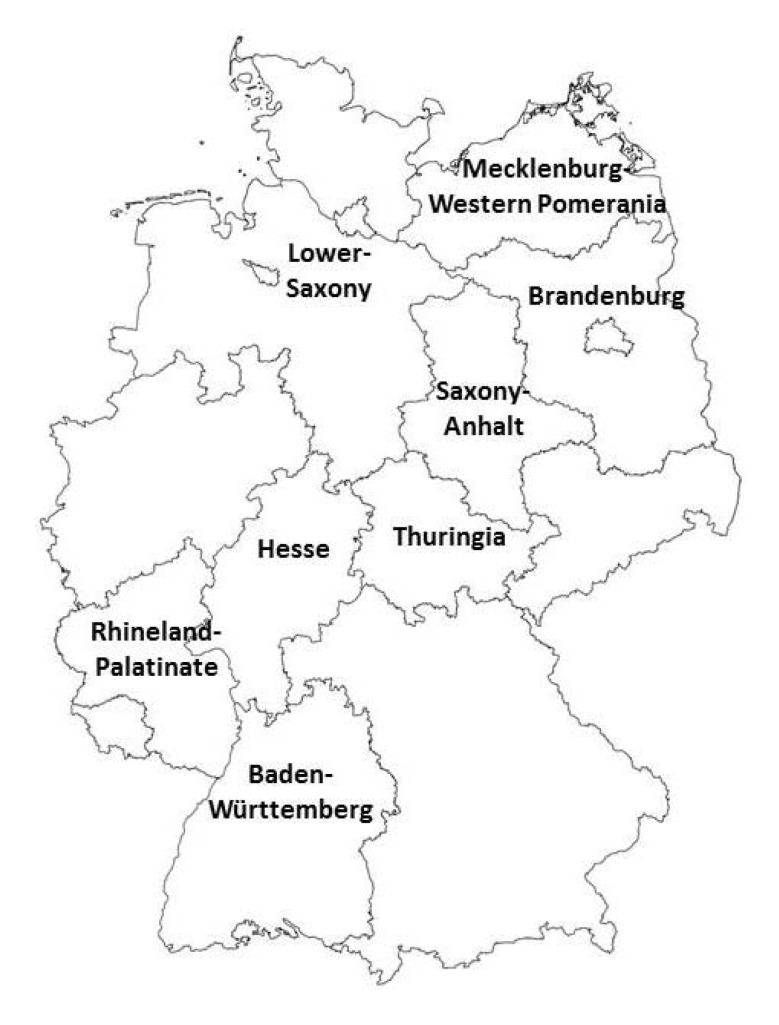
Origin of the German horse sera samples (from eight different federal states).

*Blood samples.* Sera from 5,178 horses, primarily fallen stock animals, but also from live horses in animal clinics or veterinary institutes were collected. The samples originated from eight different federal states of Germany (Figure 1). Sera were kept at −20 ºC until use.

*ELISA.* Sera were screened for WNV specific antibodies using a commercially available competition ELISA, which allowed species-independent recognition of WNV antibodies against the Pr-E envelope protein, following the manufacturer`s instructions (ID Screen^®^ West Nile Competition, IDVet, Montpellier, France). Additionally, to detect recent WNV infection in horses, a commercially available IgM capture ELISA was used (IDEXX IgM WNV Ab Test, IDEXX Europe B.V., Hoofddorp, The Netherlands).

*VNT.* ELISA results were confirmed by a virus neutralization test carried out under biosafety level 3 conditions using Vero cells on 96-well plates as described previously [23]. Test serum dilutions (20 µL starting serum material) were pre-incubated with 100 TCID_50_ of WNV strain NY 99 (lineage 1, accession no. AF196835) and/or strain Austria (lineage 2, accession no. HM015884, kindly provided by N. Nowotny, Institute of Virology, University of Veterinary Medicine, Vienna, Austria). All samples were run in duplicate and VNT titers were calculated 6 to 7 days after infection, depending on the cytopathic effects in the infected control wells. The neutralizing antibody titer, the neutralization dose 50% (ND_50_), of a serum was defined as the maximum dilution which inhibited cytopathic effects in 50% of the wells, and was calculated according to the Behrens-Kaerber method. ND_50_ values of above 10 were considered positive. TBEV neutralization test to check cross-reactivity of ELISA results was carried out following the same protocol except that the TBEV strain Neudoerfl (kindly provided by F. Hufert, Institute for Virology, Göttingen Germany; GenBank accession no. U27495) was used.

## 3. Results and Discussion

We investigated 5,178 horse sera from eight different federal states of Germany (Figure 1 and Table 1) with some serological methods. Equine sera mostly collected from horse carcasses were well suitable for testing in a commercial competitive ELISA, without producing high background reaction, which is a frequently observed phenomenon, although in some cases sample quality was quite poor.

**Table 1:** Total number of horse sera sampled in Germany from 2010 till 2012.

Federal States	Abbr.	Samples tested in year:		
		2010	2011	2012
Baden-Württemberg	BW	1	2	-
Brandenburg	BB	-	779	-
Hesse	HE	88	-	125
Lower Saxony	NI	-	79	307
Mecklenburg-Western Pomerania	MV	-	565	-
Rhineland-Palatinate	RP	-	-	669
Saxony-Anhalt	ST	-	799	1,041
Thuringia	TH	-	723	-
**in total:**	**for three years: 5,178 serum samples**			

**Table 2:** Results by commercial WNV ELISA.

Federal states abbr.	All samples tested	Negative in competitive ELISA	Reactive (positive) in competitive ELISA	Results by igm-ELISA (positive/tested)
**BW**	3	3	0	0/0
**BB**	779	779	0	0/0
**HE**	213	206	7	0/7
**NI**	386	380	6	0/6
**MV**	565	562	3	0/3
**RP**	669	656	13	0/13
**ST**	1,840	1,821	19	0/19
**TH**	723	710	13	0/13
**total**	**5,178**	**5,117**	**61**	**0/61**

### 3.1. ELISA Results

5,178 sera could be investigated by competitive WNV ELISA and 5,117 were classified as clearly negative. There was only a small proportion (n = 61) of ELISA positive WNV reagents in the study (1.17% seroprevalence, Table 2). Additionally, all 61 competitive ELISA-positive sera were investigated by the commercially available IgM ELISA to detect a recent WNV infection in horses. The IgM ELISA is very useful for epidemiological studies as well as for disease diagnosis because the presence of IgM antibodies indicates a recent infection of the host. The capture ELISA used detects specific West Nile virus IgM antibodies in horse sera, which can be detected up to 3 months after infection. In none of the 61 investigated samples WNV IgM antibodies were detected, so that there was no indication for recent infections (Table 2).

### 3.2. VNT Results

It is well-known that the commercial competitive WNV ELISA shows a high degree of cross-reactivity with other flaviviruses and positive reactions therefore need to be reconfirmed by flavivirus-specific neutralization assays. By using discrimination neutralization assays almost all reactive ELISA results could be clearly linked to the presence of specific flavivirus antibodies in these animals. The majority of positive ELISA results was clearly linked to the presence of TBEV antibodies in the respective equines (Table 3).

**Table 3:** Positive results by different VNT (neutralization titres in brackets).

Federal states abbr.	Positive comp. ELISA samples tested by VNT	Positive sera by WNV-VNT (ND_50_)	Positive sera by TBEV-VNT (ND_50_)
**BW**	0	-	-
**BB**	0	-	-
**HE**	7	1 (120)	6 (40–480)
**NI**	6	2 (1,280)	4 (80, 120, 240)
**MV**	3	*	*
**RP**	13	1 (320)	8 (20–640)
**ST**	19	1 (640), rest *	*
**TH**	13	*	*

* not evaluable because of serum toxicity for cells.

The discriminating WNV neutralization test showed only five horses with specific WNV antibodies, two from Lower-Saxony, one from Hesse, one from Saxony-Anhalt and one from Rhineland-Palatinate (Table 3). Three out of the five positive reagents in VNT were animals previously vaccinated against WNV. For the remaining two equines a previous stay in a WNV endemic area could not be excluded.

Only few TBEV cases in horses have been described to date, although infections seem to be more common. In Germany *e.g.*, a TBEV-related disease in a horse has been reported recently [22,24]. Müller *et al.* investigated a population of 240 horses in the endemic region of Marburg-Biedenkopf and identified 2.9% horses with TBEV neutralizing antibodies. Klaus *et al.* [22] described a TBEV infected horse from Bavaria with clinical signs, which was detected in routine examination. Consequently, TBEV infection should be considered as differential diagnosis for WNV infections in horses in TBEV endemic areas. Our results are in accordance with a similar study in Austria, where an unexpectedly high infection rate with TBEV was identified in a large herd of horses [19]. The eight horses from Rhineland-Palatinate originated from defined TBE risk areas, which might explain the positive WNV-ELISA and TBEV-VNT results. The positive results in seven horses from Hesse were in accordance with the epidemiological TBEV situation in this area [25]. The districts Lahn-Dill, Kassel, Frankfurt and Gießen have a well-known TBEV-history with the occurrence of human cases (Lahn-Dill-Kreis n = 3; Kassel n = 1; Frankfurt n = 4; Gießen n = 1) between 2002 and 2012. Furthermore, the TBEV endemic region of Marburg-Biedenkopf, which was described by Müller *et al.* 2006, directly abuts on these districts. Of the TBEV positive equine sera from Lower Saxony (Table 3), three sera originated from horses coming from areas where also human cases had been reported in the past. One horse originated from Neustadt am Rübenberge, a city in the vicinity of Hannover, and two others from Uetze, a community in the Hannover region, where three indigenous human TBEV cases each occurred in 2009 and 2011, one human case was observed in 2010 and four human cases in 2012 [25]. The remaining seropositive horse was a recently imported animal from Poland, for which a TBEV exposition at its origin cannot ultimately be excluded. Interestingly, all TBEV antibody positive animals showed no clinical signs of encephalitis or relevant symptoms.

Unfortunately, the etiology of the remaining four sera from Rhineland-Palatinate (positive WNV ELISA results, but negative in all conducted VNT, Table 3) could not be resolved. There was also no evidence of cross-reactivity with Usutu-virus (USUV), since all four sera were negative by an USUV neutralization test (using German USUV strain BH65/11-02-03). Furthermore a small number of the WNV ELISA positive sera (especially from MV, ST and TH) had to be excluded from the VNT because of serum toxicity for cells. However, all these serum samples were also negative by ELISA for WNV IgM antibodies, so that there was no indication for recent WNV infections in any of the horses monitored in the here presented study. Taken together, these ELISA and VNT results suggest that there are no active WNV infections in horses in Germany at presence. The here presented results are in agreement with those of a previous study [17].

Our study shows that equines can be useful indicators to detect the occurence of WNV in an area. However, these sentinels work only in areas without TBEV history and WNV vaccination (WNV infection antibodies cannot be discriminated from vaccination-derived antibodies). In order to directly reveal TBEV cross-reactions it may be helpful to use a TBEV-antibody ELISA [26] in parallel. The use of this assay in horses may even be useful to reveal TBEV endemic areas.

## 4. Conclusions

We have successfully used a sequential WNV IgG ELISA, WNV IgM ELISA, discriminatory VNT and TBEV ELISA approach to conduct a WNV monitoring study using equine blood samples drawn from sick animals in horse clinics, from healthy horses in stables and collected from carcasses at rendering plants. Apart from occasional TBEV infections in horses, no WNV infections in horses that perished from 2010 to 2012 were found. Cross-reacting TBEV antibodies in ELISA must be reconfirmed by flavivirus-specific neutralization assays. According to these data, there is no evidence for indigenous WNV infections in horses in Germany so far.
